# Attentional bias induced by stimulus control (ABC) impairs measures of the approximate number system

**DOI:** 10.3758/s13414-020-02229-2

**Published:** 2021-01-18

**Authors:** Marcus Lindskog, Leo Poom, Anders Winman

**Affiliations:** 1 Department of Psychology, Uppsala University, P.O. Box 1225, SE-751 42 Uppsala, Sweden; 2 Department of Education, Uppsala University, Uppsala, Sweden

**Keywords:** Approximate number system, Attention, Numerosity judgments, Eye-tracking, Stimulus control, Numerical cognition, Mathematical performance

## Abstract

Pervasive congruency effects characterize approximate number discrimination tasks. Performance is better on congruent (the more numerous stimulus consists of objects of larger size that occupy a larger area) than on incongruent (where the opposite holds) items. The congruency effects typically occur when controlling for nonnumeric variables such as cumulative area. Furthermore, only performance on incongruent stimuli seems to predict math abilities. Here, we present evidence for an attentional-bias induced by stimulus control (ABC) where preattentive features such as item size reflexively influence decisions, which can explain these congruency effects. In three experiments, we tested predictions derived from the ABC. In Experiment 1, as predicted, we found that manipulation of size introduced congruency effects and eliminated the correlation with math ability for congruent items. However, performance on incongruent items and neutral, nonmanipulated items were still predictive of math ability. A negative correlation between performance on congruent and incongruent items even indicated that they measure different underlying constructs. Experiment 2 demonstrated, in line with the ABC account, that increasing presentation time reduced congruency effects. By directly measuring overt attention using eye-tracking, Experiment 3 revealed that people direct their first gaze toward the array with items of larger individual size, biasing them towards these arrays. The ABC explains why the relation between performance on approximate number discrimination tasks and math achievement has been fragile and suggests that stimulus control manipulations have contaminated the results. We discuss the importance of using stimuli that are representative of the environment.

Humans are believed to be equipped with an approximate number system (ANS) that can represent the number of objects in a visual scene without the aid of symbols (Feigenson, Dehaene, & Spelke, 2004). The ANS is imprecise, with representations becoming noisier as the number of objects in a scene increase. Importantly, there are considerable individual differences in the precision, or acuity, of the ANS (e.g., Halberda, Ly, Wilmer, Naiman, & Germine, 2012), which are related to mathematical ability in both children (e.g., Halberda, Mazzocco, & Feigenson, 2008; Schneider et al., 2017) and adults (Chen & Li, 2014; Price, Palmer, Battista, & Ansari, 2012). Those with a more precise ANS tend to be better at mathematics. The triple code model (Dehaene, 1992) of numerical abilities gives one possible explanation for the relation. According to the model, the ANS acts as the primary semantic representation of numbers onto which both verbally presented number words and visually perceived Arabic digits are mapped. Thus, people with a more precise ANS can more effortlessly understand numbers and acquire mathematics through formal education. However, not all studies have been able to document a relation between ANS acuity and mathematical ability, which has sparked a debate concerning the reason for the mixed results (Inglis, Attridge, Batchelor, & Gilmore, 2011).

When judging how many objects are in a visual scene, people could potentially rely on visual cues other than numerosity (see, e.g., Gebuis & Reynvoet, 2011, for an overview). For example, if two stimulus sets contain objects of the same shape and size (with object size, we here refer to the area covered by an individual item), the more numerous stimulus set will also have a larger cumulative area occupied by the objects (and convex hull if object density is kept constant or higher density if convex hull is kept constant, where convex hull is the smallest convex polygon that contains a set of dots). Although this is a necessary consequence of the physical state of affairs, it has been considered a problem. To address the issue of dependency between number and visual cues, a common procedure has been to equate cumulative area on half of the trials and equate average object size on the other half (e.g., Halberda & Feigenson, 2008; Piazza, Pica, Izard, Spelke, & Dehaene, 2013; Sasanguie, Defever, Van den Bussche, & Reynvoet, 2011).

However, it is not possible to equate stimuli for all visual cues at the same time. Manipulating one visual cue, while keeping numerosity fixed, will necessarily affect other cues. For example, equating two stimulus sets of differing numerosity on their cumulative area will result in objects in the more numerous stimulus set being smaller than objects in the less numerous one. Accordingly, people could then instead use object size as a cue to decide which is the more numerous stimulus set. We refer to the above control method as a *partly congruent* design, in which some, but not all, visual cues covary with numerosity. With a partly congruent design, the more numerous stimulus set thus consists either of smaller objects (when controlling for area), or of objects occupying a larger cumulative area (when controlling for size), but the more numerous stimulus set never consists of objects with larger size.

Another approach to solving the problem of dependency between number and visual cues has been to pit visual cues against numerosity in a much more conspicuous way (e.g., DeWind & Brannon, 2012; Gilmore et al., 2013). With this approach, stimuli are created such that visual cues either covary positively or negatively with numerosity. For the former stimuli, the more numerous stimulus set has a *larger* convex hull, a larger cumulative area, and a larger average size of the objects. In contrast, for the latter, the more numerous stimulus set has a *smaller* convex hull, a smaller cumulative area, and a smaller average size of the objects. Stimuli in which individual objects of larger size are also more numerous are often referred to as *congruent* stimuli in contrast to *incongruent* stimuli, where the more numerous objects are smaller. We refer to this control method as the *full congruent/incongruent* design. Note that because there is no apparent reason why objects of larger individual size, in general, are more numerous than smaller objects, most natural scenes will be partly congruent, with full congruent/incongruent scenes occurring as exceptions. Empirically, studies have found both a larger-is-more bias (e.g., Gilmore et al., 2013; Hurewitz, Gelman, & Schnitzer, 2006) and a smaller-is-more bias (e.g., Ginsburg & Nicholls, 1988; Miller & Baker, 1968; Poom, Lindskog, Winman, & van den Berg, 2019; Tokita & Ishiguchi, 2010) in numerosity tasks. The reason for the inconsistent results is unclear, but the magnitudes in the differences in item sizes used could play a role. We have previously argued against using stimuli with a full congruent/incongruent design because this procedure might induce processes that would not otherwise influence participants’ responses by making perceptual cues salient (Lindskog, Winman, Juslin, & Poom, 2013). There is no standard method of stimulus control. Clayton, Gilmore, and Inglis (2015) tested the same participants on ANS tests with the two most commonly used control schemes and showed that performance on one test only explained 7% of the variance in the other, suggesting that these tests measure different psychological constructs.

Several studies (e.g., Gilmore et al., 2013; Szűcs, Nobes, Devine, Gabriel, & Gebuis, 2013) using the full congruent/incongruent design have found strong congruency effects; performance is considerably lower with incongruent than with congruent stimuli. An extreme example of the congruency effect is found in the study by Szűcs et al. (2013), where participants even performed below chance on incongruent stimuli. Accuracy was so poor for incongruent stimuli (mean Weber fraction above 7) that performance would be considered as pathological by normative standards of this measure (e.g., Weber fraction of .36, Mazzocco, Feigenson, & Halberda, 2011, and .36, Piazza et al., 2013, have been reported for children with dyscalculia). Apparently, participants’ judgments in this study were guided by something else than a system dedicated to extracting abstract numerosity. Strong congruency effects have accordingly been used as evidence that humans lack a dedicated system for number processing altogether, but instead integrate visual cues to inform numerosity (Gebuis & Reynvoet, 2012; Leibovich, Katzin, Harel, & Henik, 2017).

It has been suggested (Gilmore et al., 2013; Szűcs et al., 2013) that the incongruent/congruent distinction could be viewed in analogy with a Stroop task, where participants fail to ignore a task-irrelevant dimension: “on incongruent trials, participants need to inhibit a response based on these salient characteristics and respond only to the number of dots” (Gilmore et al., 2013, p. 2). In line with the Stroop analogy, domain-general executive functions such as inhibitory control have been argued to play an essential role in numerosity tasks that apply the full congruent/incongruent design. Szűcs et al. (2013), for example, speculated that the lower performance of children than adults on numerosity tasks might be due to the poorer inhibitory control of children. Gilmore et al. (2013) took this argument one step further and proposed that “it is the inhibition element, rather than the precision of numerical representations element, that results in a significant correlation with mathematics achievement” (p. 7). In support of this, the authors showed that performance on incongruent, but not on congruent, items predicted math achievement (see, e.g., Fuhs & McNeil, 2013; Norris & Castronovo, 2016, for replications and similar interpretations). They also demonstrated that the correlation between ANS acuity and math achievement disappeared when controlling for inhibitory control, a finding the authors suggested might generalize to adults. This finding, together with previous research indicating that inhibitory control predicts mathematics achievement (e.g., Bull & Lee, 2014), suggests that the relation between ANS acuity and math performance might be spurious and caused solely by the inclusion of incongruent items in ANS acuity tasks. Below, we refer to this alternative as the *inhibition hypothesis.* Challenging this hypothesis, Castaldi and colleagues (Castaldi, Mirassou, Dehaene, Piazza, & Eger, 2018) showed that dyscalculics were strongly affected by an unattended dimension when judging number, but performed comparable to control subjects when judging size. These findings suggest, as the authors argued, that the influence of nonnumeric dimensions on number judgements is not due to a domain-general inhibition, but to a heuristic strategy of coping with a weak approximate number system (Castaldi et al., 2018).

The inhibition hypothesis, if shown to be true, could have a dramatic impact on the entire research area, results being contrived artifacts caused by attempts at stimulus control in the laboratory. Indeed, a majority of studies have been motivated by the intriguing theoretical and applied possibilities implied by the correlation between ANS acuity and math performance.

## The present study: The attentional bias induced by control (ABC) hypothesis

In the present paper, we present a framework, the attentional bias induced by stimulus control (ABC) hypothesis, that can explain several of the intriguing phenomena described above: Why is the correlation between measures of ANS acuity and math achievement so fragile and vary from study to study? What is the cause of persistent congruency effects? How is the correlation with math achievement affected by congruency effects? Under which circumstances do congruency effects deflate or nullify this correlation, and when do they not? Below, we first outline the ABC hypothesis and its underlying assumptions. We then derive predictions that are tested in three empirical studies.

Researchers have put much effort into controlling for nonnumeric variables, such as cumulative area and the convex hull when measuring the ability to decide which of two stimulus sets is the more numerous. While these efforts towards experimental control in one sense are commendable, they have had unintended effects. The objective has been to ensure that people do not rely on any nonnumeric visual cues when estimating numerosity. We propose, however, that the consequence of this experimental control is that participants’ attention is instead *directed* towards the very variables researchers intended to make uninformative. This, in turn, gives rise to a bias, which would probably have a marginal impact on judgments had the control not been in place. Put differently, stimulus control induces differences in saliency between the to be compared arrays, which leads to a rapid, involuntary directing of attention towards the more salient stimulus set. Thus, according to the ABC hypothesis, congruency effects found in previous research are the result of fast bottom-up driven attentional processes. In line with this notion of a bias, Odic and Halberda (2015) showed in a recent eye-tracking study that participants during an ANS acuity task tend to choose the stimulus they first focus on.

How come participants’ attention becomes directed towards the cues that are intended to be uninformative? Visual attention is guided by *preattentive features* that reflexively direct attention to a region of a scene (Treisman & Gelade, 1980). Among the features that have been shown to have this guiding function is the number of objects in a scene, which seems to spontaneously attract attention in humans (e.g., Castaldi, Burr, Turi, & Binda, 2020; Cicchini, Anobile, & Burr, 2016; Ferrigno, Jara-Ettinger, Piantadosi, & Cantlon, 2017). Other attention-grabbing features are object size, colour, motion, and orientation (Wolfe & Horowitz, 2004). These features are preattentive in the sense that they are available to the visual system before the actual act of selection. If neighbouring stimuli differ on a preattentive dimension, this difference will guide attention in an involuntary bottom-up manner. The preattentive dimension object size varies routinely between stimulus sets in visually controlled tasks, thus potentially competing with number for attention.

When considering *congruent* and *incongruent* stimuli, only the latter has been shown to predict math achievement. As discussed above, this has been interpreted to indicate that incongruent stimuli are analogous to a Stroop task that makes them measure inhibition rather than ANS acuity (Gilmore et al., 2013). The Stroop analogy is, however, incomplete. The original Stroop task involves a conflict between a well-practiced, automatized task (reading) and a controlled task (color naming). For incongruent stimuli, there is no automatized process, and it is not clear a priori why certain variables would compete with judgments of number. For example, the Stroop analogy implies that objects of larger individual size appear to be more numerous. However, in contrast with this prediction, Poom et al. (2019) showed, with a method that excludes rapid attention effects, that objects of larger individual size instead are perceived as less numerous.

The ABC hypothesis suggests a different explanation for the discrepancy between performance on congruent and incongruent stimuli and math achievement, purely based on uncontroversial, psychometric measurement considerations. High performance with congruent stimuli could indicate either that the individual has an acute ANS or that his or her judgments are influenced by nonnumeric cues. This would be particularly problematic if, as suggested, nonnumeric cues are used strategically to a differential degree by individuals to cope with a weak number sense (Castaldi et al., 2018). This renders congruent, unlike incongruent, items invalid measures of ANS acuity. These measures are calculated from pooled performance on congruent and incongruent stimuli. Accordingly, they are affected by attentional processes. Furthermore, half of the items that determine performance cannot be considered to measure ANS acuity, resulting in a contaminated measure. According to the inhibition hypothesis (e.g., Gilmore et al., 2013), it is the incongruent items that invoke the correlation between ANS acuity and math. According to our explanation, it is, on the contrary, the congruent items that deflate this correlation through attentional bias.

Because there is no standard method of stimulus control, measurements of ANS have been more or less impure. We hypothesize that there is a causal link between ANS acuity and math performance, but that results in terms of correlations with math achievement in previous research have been inconsistent due to this varying degree of contamination of ANS measures. Most previous studies have used controls for nonnumeric visual cues. However, at least one previous study (Lindskog et al., 2013) has, in line with this expectation, indicated that ANS performance measured with unconstrained stimuli (without control of nonnumeric properties) predicts math performance.

In the present study, we investigate the extent to which preattentive features influence performance on a task designed to measure the acuity of the approximate number system, and the subsequent link to math performance. The ABC hypothesis makes the following predictions:

1. A test of number estimation that eliminates stimulus differences between sets of objects on a prominent preattentive dimension such as size will promote unbiased initial attention to both sets of objects and thereby be a valid measure of ANS acuity. Accordingly, performance on this test will predict math-related variables. The inhibition hypothesis implies the diverging prediction that using such a test should eliminate the correlation
2. Introducing a difference between stimuli on a preattentive dimension (such as size) will bring about attentional factors, contaminating the measure. Accordingly, the full set of stimuli will be less predictive of math-related variables than a subset without a difference in the preattentive dimension.
3. Within a test designed to measure ANS acuity in which stimuli differ on the preattentive dimension, the incongruent subset will be a better predictor of math-related variables than the congruent subset. It follows from Predictions 2 and 3 that this type of test will exhibit poor internal consistency (poor correlation between performances on the test halves) because the two halves of the test partly measure different things.
4. Prolonged stimulus exposure will reduce attentional bias. Because this bias is the cause of congruency effects, they will also be reduced.
5. With a direct measure of attention, on a stimulus set relevant to visual control in prior research, eye-tracking will disclose rapid initial attention directed towards the more numerous stimulus set for congruent stimuli and towards the less numerous stimulus set for incongruent stimuli.

Predictions 1–3 are tested in Experiment 1, Prediction 4 is tested in Experiment 2, and Prediction 5 is tested in Experiment 3.

## Experiment 1

In Experiment 1, participants completed two dot-comparison tests designed to measure ANS acuity. In the *neutral* test, we constrained the average dot size to be the same in both arrays, but imposed no other controls. Accordingly, as would be expected in the natural environment, cumulative area, convex hull, and density were allowed to covary positively with numerosity. In contrast, in the *size-manipulated* test, we used a method analogous to the full congruent/incongruent design, resulting in the more numerous stimulus set consisting either of larger or smaller objects than the less numerous stimuli. We also measured participants’ arithmetic fluency, self-ratings of math proficiency, and math anxiety. In the neutral test, with little expected attentional bias towards a particular stimulus, performance should be in line with previous research (Chen & Li, 2014).

In contrast, if manipulating the preattentive dimension size by congruent/incongruent stimulus sets introduces strong attentional processes that dominate the extraction of numerosity from the two arrays, we expect this correlation to be deflated. The size-manipulated test can, in turn, be divided into a *congruent* and an *incongruent* subset. We performed analyses separately on these subsets, expecting to replicate previous findings (e.g., Gilmore et al., 2013) with an attenuated correlation in the congruent subset, but not in the incongruent subset. We also expected performance with the congruent subset to be less predictive of performance with the neutral test than performance with the incongruent subset.

### Method

#### Participants

Participants (20 males, 38 females, *M*_age_ = 24.1 years, *SD*_age_ = 5.8 years) were mainly undergraduate students. All gave informed consent to participate and received either two cinema vouchers or course credit for participating.

#### Procedure and materials

The study included tests of individual differences of arithmetic fluency and ANS acuity. The total time of the experiment was 90–120 minutes. Tasks were PC administered individually in separate one-person cubicles.

##### Math-related variables

The arithmetic fluency task (adopted from Gebuis & van der Smagt, 2011) consisted of four separate tests of addition, subtraction, multiplication, and division. A participant was given 150 seconds per test (a total of 10 minutes) to complete as many arithmetic problems as possible. Problems increased in difficulty within the set. Arithmetic fluency was defined as the number of correctly answered problems. We calculated both a combined score for all four arithmetic operations, as well as scores for each operation and the proportion of attempted tasks solved correctly, in order to get a measure of calculation precision in addition to speed. We used two self-reported variables. Participants rated their view of their own proficiency at mental arithmetic without pen and paper. They also completed a short questionnaire, measuring their *math anxiety* (AMAS; Hopko, Mahadevan, Bare, & Hunt, 2003). In addition, we use a composite math measure based on average standardized scores on arithmetic fluency and self-report measures.

##### The numerosity comparison tests

The stimuli were presented using MATLAB (The MathWorks, Friedrichsdorf, Germany). A fixation cross appeared in the center of the screen 500 ms prior to the 300-ms stimulus presentation. The presentation time was too short to allow counting. Stimuli consisted of blue and yellow dots of different numerosities appearing on a grey background. The total number of dots presented in the different stimuli varied between 14 and 30. Dot sizes were drawn from a uniform distribution on the range [0.35, 0.70] degrees of visual angle (see Table 1 for further details about stimulus set characteristics). The two stimulus sets were displayed side by side and did not overlap. Each stimulus set subtended an area of 13 × 13 visual degrees, and the entire stimulus subtended an area of 26 × 13 visual degrees (the width and height of the entire stimulus were 792 and 406 pixels, respectively). Participants were given four practice trials. They were instructed to determine which color was the more numerous by pressing a color-coded key. ANS acuity is measured as the proportion of correct answers. The two tests described below were blocked and presented in a counterbalanced order over participants. Each test consisted of 120 trials.

**Table 1:** Stimulus characteristics for each experiment and stimulus type

Experiment	Stimulus type	Stimulus property							
		*N*				Ratio			
		Min	Max	Total min	Total max	*N*	Total area	Average area	Convex hull
Exp 1	Congruent	7	16	15	30	1.14	2.24	1.96	1.11
		10	12	22	22	1.20	2.35	1.96	1.13
		6	16	14	28	1.33	2.62	1.96	1.18
	Incongruent	7	16	15	30	1.14	0.58	0.51	0.96
		10	12	22	22	1.20	0.61	0.51	1.05
		6	16	14	28	1.33	0.68	0.51	1.03
	Neutral	7	16	15	30	1.14	1.14	1.00	1.06
		10	12	22	22	1.20	1.20	1.00	1.09
		6	16	14	28	1.33	1.34	1.00	1.11
Exp 2	Congruent	7	16	15	30	1.14	6.56	5.74	2.08
		5	12	11	22	1.20	7.08	5.90	2.18
		6	16	14	28	1.33	7.37	5.53	2.34
	Incongruent	7	16	15	30	1.14	0.39	0.34	0.61
		5	12	11	22	1.20	0.40	0.33	0.59
		6	16	14	28	1.33	0.44	0.33	0.62
Exp 3	Congruent	7	16	15	30	1.14	6.29	5.50	2.00
	Incongruent	7	16	15	30	1.14	0.37	0.32	0.55
	Neutral	7	16	15	30	1.14	1.14	1.00	1.06

*Note.* For all the ratio properties, the ratio was calculated by dividing the property of the more numerous set with that of the less numerous set

**Fig. 1:**
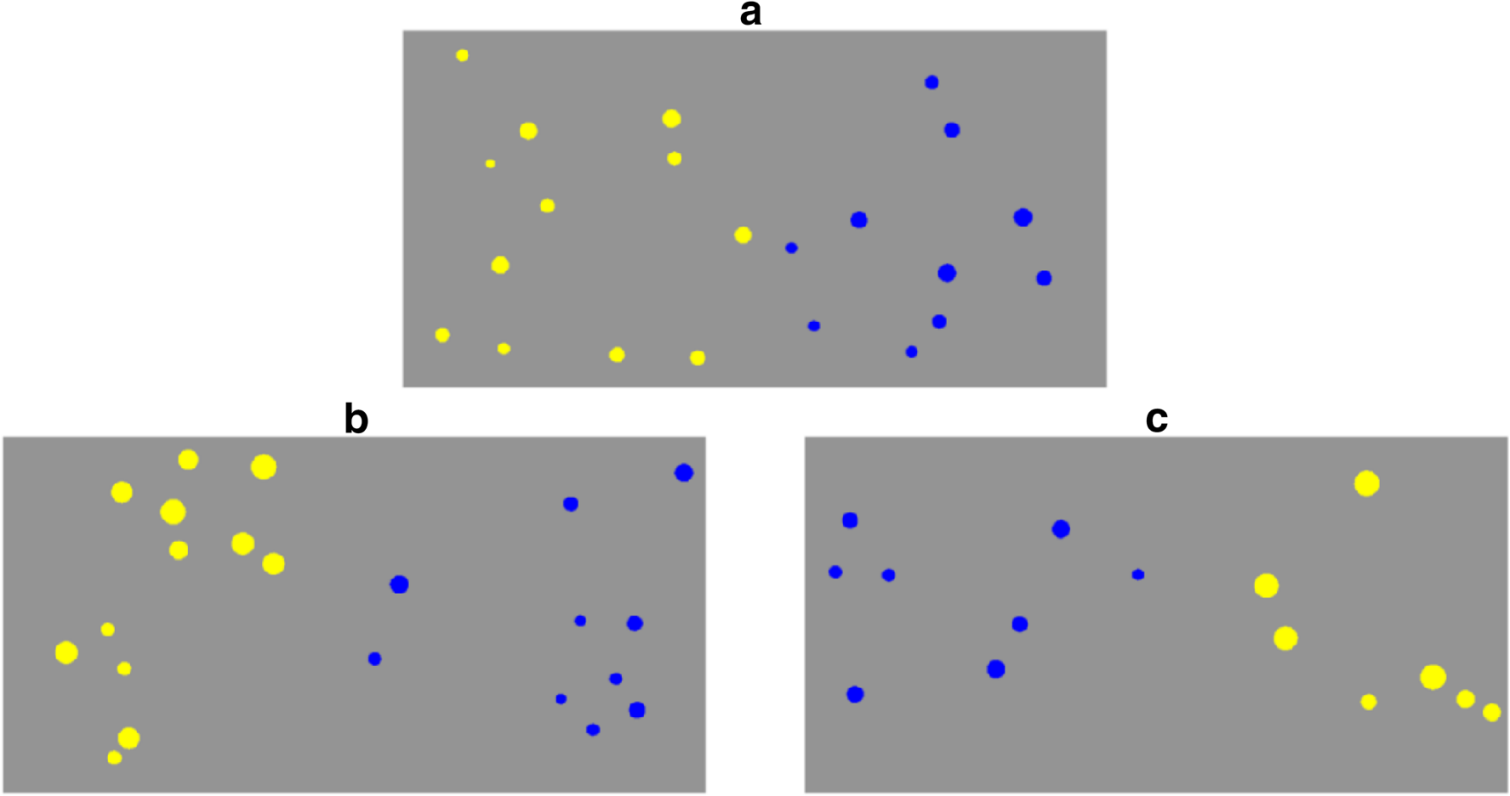
Illustration of stimuli in the three conditions of Experiment 1. **a** A neutral stimulus. **b** A congruent stimulus. **c** An incongruent stimulus

For the *neutral* test, we created 60 stimuli in three difficulty conditions (20 in each) with ratios between the two to be compared numerosities of 3:4, 5:6, and 7:8. Dots in the two stimulus sets varied randomly in size, but were on average equally large (see Fig. 1a). Because dots had the same average size, cumulative area covaried positively with numerosity. For the *size-manipulated* test, we split the stimuli into two subsets, equally distributed over stimulus ratios. In the *congruent subset*, we multiplied the dot radii with 1.4 in the more numerous stimulus (see Fig. 1b), while in the *incongruent subset*, the radii of the dots in the less numerous stimulus were subjected to this operation (see Fig. 1c). Accordingly, in the congruent subset, the more numerous stimulus consisted of larger dots, whereas the opposite holds for the incongruent subset. The size-manipulated test is analogous to (rather than an exact replication of a particular control) the method of controlling for visual cues used in many previous studies, by which size covaries with numerosity in different directions for congruent/incongruent items.

## Results

On the neutral test, the proportion of correct answers was .75 (*s* = .06).^1^ In the size-manipulated test, there was a congruency effect, with better performance in the congruent (prop. correct = .78, *s* = .13) than in the incongruent (prop. correct = .65, *s* = .15) subset, replicating previous findings, *t*(57) = 3.85, *p* < .001. The overall correlation between performance on the neutral and size-manipulated tests was significant, *r*(56) = .50, *p* < .001. We investigated to what degree items in the two subsets of the size-manipulated test measure the same underlying psychological construct, as desirable from a psychometric perspective, by calculating the Pearson correlation (two-sided) between performance on the two subsets. For all correlations below, we also calculated a Bayes factor in JASP (JASP Team, 2020) using the default stretched beta prior width = 1 (i.e., all correlations between −1 and +1 are given an equal prior probability). The Bayes factor BF_10_ is the ratio between the probabilities of the results given H_1_ and H_0_ and is thus a measure of the relative evidence in favour of H_1_. Bayes factors have several other advantages (Wagenmakers et al., 2018). For example, unlike the p value, the Bayes factor can be interpreted as strong evidence in favour, or against, the null hypothesis There was a strong significant *negative* correlation between performance on the two subsets, *r*(56) = −.69, *p* < .001, *BF*_*10*_ = 5.1 ∙ 10^6^.^2^ Test performance on the incongruent subset was not related significantly to the total score of the size-manipulated test, *r*(56) = .11, *p* = .41, *BF*_*10*_ = .23,^3^ whereas test performance on the congruent subset was, *r*(56) = .65, *p* < .001, *BF*_*10*_ = 3.8 × 10^5^. Performance on the congruent subset, on the other hand, was not predictive of performance in the neutral test, *r*(56) = .11, *p* = .39, *BF*_*10*_ = .23, whereas performance on the incongruent subset was, *r*(56) = .33, *p <* .05, *BF*_*10*_ = 3.6. Thus, performance on congruent stimuli was not indicative of participants’ ability to estimate number with a test in which stimuli have not been size-manipulated.

Next, we turn to the question of the relation between performance on the two tests, and math performance. Table 2 shows the zero-order Pearson correlations between performance on the neutral and size-manipulated tests, respectively, and math-related variables.

**Table 2:** Pearson correlations, two-sided, (*r*) with Bayes Factors (BF_10_) between performance in neutral and size manipulated tests, and the congruent and incongruent subsets of the size-manipulated test, and math-related measures

Condition		Mathematics related measure								
		MC	AF	MP	ADD	SUB	MUL	DIV	MA	M-ab
Neutral	*r*	.50***	.41***	.53***	.41**	.43***	.39***	.19	−.33**	.26**
	*BF*_*10*_	466.65	21.68	1117.98	22.06	41.93	14.26	0.43	3.39	1.11
Size manipulated	*r*	.16	.15	.23	.18	.22	.05	.08	−.20	.01
	*BF*_*10*_	0.34	0.31	0.73	0.38	0.62	0.18	0.19	0.52	0.16
Congruent	*r*	−.09	−.06	−.10	.03	−.05	−.15	−.05	.07	−.04
	*BF*_*10*_	0.20	0.18	0.22	0.17	0.18	0.30	0.18	0.19	0.17
Incongruent	*r*	.27**	.23*	.36**	.12	.28**	.24*	.14	−.28**	.06
	*BF*_*10*_	1.32	0.69	6.46	0.25	1.38	0.87	0.29	1.53	0.18

*Note*. *MC* math composite measure, *AF* arithmetic fluency, *MP* proportion of attempted problems solved; *ADD* addition, *SUB* subtraction, *MUL* multiplication, and *DIV* division problems solved, *MA* math anxiety; M-ab = self-reported mathematical computational ability. *p < .10, **p < .05, ***p < .001

Performance on the neutral test correlated significantly with eight out of nine math-related measures, with division being the exception. Replicating previous research (Lindskog, Winman, & Poom, 2017), performance in this test was also negatively correlated with math anxiety. In contrast, performance on the size-manipulated test was not significantly correlated with any of the math-related measures.

Table 2 also shows zero-order Pearson correlations between performance on the congruent and the incongruent subset (of the size-manipulated test) and math-related variables. Performance on the congruent subset was not correlated with any math variable, whereas performance on the incongruent subset correlated positively with all eight math performance measures, and significantly so with the composite math measure, the proportion of problems solved, and subtraction and negatively with math anxiety. There were also positive correlations with *p* < .10 for arithmetic fluency and multiplication for this subset, although no support for a correlation was supported for these cases by Bayes factors.

### Discussion

Participants performed both a neutral test, without control for visual cues and a test in which item size was related to numerosity by use of congruent and incongruent stimuli. Within subjects, ANS acuity both predicted and failed to predict math performance depending on which index of ANS acuity that was chosen. Manipulation of item size, in a manner highly analogous to how researchers almost without exception control for nonnumeric variables, disrupted the correlation between measures of ANS acuity and math-related variables. This suggests that control for nonnumeric variables in previous research may have contributed to an underestimation of the effect size in the environment and to the fickle nature of the correlation, where a number of studies fail to replicate the finding of a correlation. Furthermore, performance on the congruent subset failed to predict ANS acuity, as indexed by the total score in the neutral test, whereas performance on the incongruent subset did.

We found a negative correlation between performance on the congruent and incongruent subsets. This goes against psychometric principles, which imply that items that measure the same construct should be positively correlated (i.e., internal consistency). This finding is consistent with the measurement-related argument in that performance on the congruent subset is not a valid measure of participants’ intuitive ability to rapidly estimate numerosity, but is confounded with a tendency to direct attention to nonnumeric variables. One possible explanation for the negative correlation is that there are individual differences in the extent to which participants are influenced by attention and visual cues, possibly due to strategies used to cope with a weak number sense (Castaldi et al., 2018). For example, some participants could be more influenced by attention (boosting performance in congruent trials) and less by a smaller-is-more bias, while others exhibit the opposite pattern (boosting performance in incongruent trials).

We propose that one candidate process that might come to dominate the extraction of numerosity when using incongruent stimuli is attention. Likely, an effect of attention on performance when using incongruent stimuli appears rapidly and early in visual processing (cf. Castaldi et al., 2020). During short presentation times (often around 300 ms) when measuring ANS acuity, the nonnumeric properties of incongruent stimuli draw people’s attention to features that are irrelevant for solving the task. Thus, if attentional processes influence performance, it might be possible to offset these processes by allowing more time to override the initial bias inflicted by attention to particular stimulus properties. We designed Experiment 2 to test this prediction.

## Experiment 2

The finding of congruency effects could be explained by assuming that there is no dedicated system for estimating numerosity (Gebuis & Reynvoet, 2012), but that people instead use other perceptual cues, such as size and area, to estimate number. However, as argued above, our account of congruency effects is that they occur due to preattentional processes induced by perceptual cues such as object size. We propose that more salient (e.g., objects of larger individual size) stimuli rapidly attract attention through bottom-up attentional processes (Wolfe & Horowitz, 2004). These processes, in turn, bias decisions. The effect of attention may have been inflated in research by the commonly used very brief presentation time (e.g., 300 ms), whereby participants after shifting their gaze towards one set of stimuli will not have time to shift focus to the other stimulus set. If the natural mode of numerosity estimation is through surrogate nonnumeric visual cues, it seems plausible that increasing presentation time (but keeping it short enough to prevent serial counting) will allow participants to build up a more stable and accurate percept of the stimuli, and therefore, by reducing noise in the input, increase congruency effects. However, if congruency effects are caused by attentional processes, the visual system should be able to counteract the initial rapid attention processes using corrective input from additional gaze shifts and thus be less susceptible to influences of nonnumerical visual cues.

In Experiment 2, we manipulated presentation time and congruency within subjects. The latter was done with a standard procedure shown to produce strong congruency effects (Gebuis & Reynvoet, 2011). Under our ABC hypothesis, we predicted an interaction between congruency and presentation time in terms of reduced congruency effects with a longer presentation time.

### Methods

#### Participants

Participants (21, five males, 16 females, *M*_age_ = 26.4 years, *SD*_age_ = 9.3 years) were mainly undergraduate students. All gave informed consent to participate and received either a cinema voucher or course credits for participating.

#### Materials and procedure

Participants individually carried out a computerized dot-comparison task that was PC administered. They were tested in separate one-person cubicles, and the total time of the experiment was approximately 60 minutes.

##### The numerosity comparison tests

The stimuli were presented using MATLAB (The MathWorks, Friedrichsdorf, Germany). We used the script provided by (Gebuis & Reynvoet, 2011), with a slight modification, to generate the stimuli. This script manipulates the size of the individual dots and the area in which the dots can appear (Gebuis & Reynvoet, 2011). Because we were primarily interested in stimuli that were fully congruent and fully incongruent, we modified the script to include only such trials. Thus, half of the trials were *congruent,* with the more numerous stimulus set consisting of larger dots (average radii 2.5 times larger) and a larger convex hull, while half of the trials were *incongruent,* with the less numerous stimulus set consisting of larger dots (average radii 1.8 times larger) and a larger convex hull (see Table 1 for further details about stimulus set characteristics). Participants were first presented with a fixation cross for 700 ms, followed by two spatially separated arrays of blue and yellow dots presented on a grey background for either 300 ms or 2,000 ms. They were instructed to indicate, with the press of a color-coded key, which of the two arrays was the more numerous. The total number of dots presented in the different stimuli varied between 11 and 30, and we used three ratios between the number of dots in the two arrays (3:4, 5:6, and 7:8).

We varied the presentation time of the two arrays within subjects to 300 ms and 2,000 ms. Both of these presentation times are too short for the two arrays to be serially counted. Participants completed 420 trials in each condition. The order of the conditions was counterbalanced.

## Results

We investigated the effects of presentation time and stimulus congruency (congruent / incongruent) on the proportion of correct responses with a two-way, within-subjects analysis of variance (ANOVA), with presentation time and congruency as independent variables, and proportion correct as the dependent variable.

**Fig. 2:**
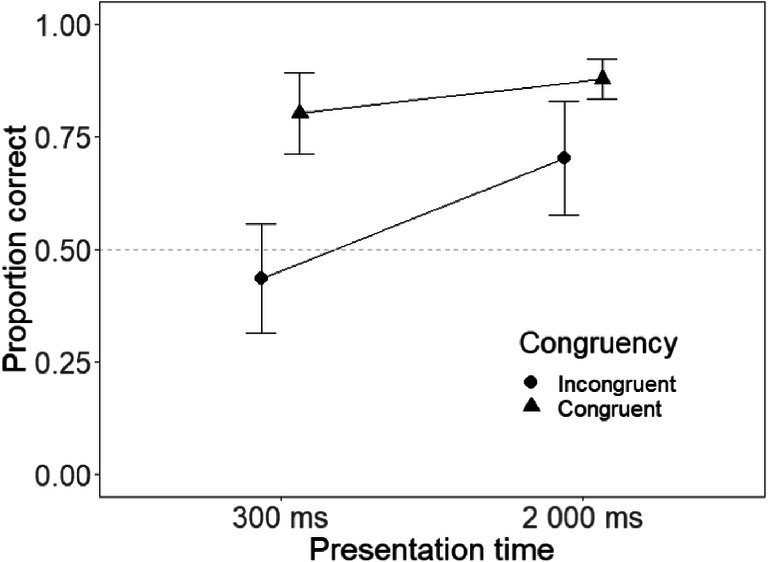
Proportion correct as a function of presentation time and stimulus congruency. Whiskers denote 95% confidence intervals, and the dashed grey line denotes chance performance at .5

Replicating previous congruency effects, the analysis showed a significant effect of stimulus congruency, *F*(1, 19) = 19.8, *p* < .001, η_p_^2^ = .51. Participants performed better in the congruent (*M* = .84, *SE* = .04) than in the incongruent condition (*M* = .57, *SE* = .04). There was also a main effect of presentation time, *F*(1, 19) = 65.4, *p* < .001, η_p_^2^ = .78, with better performance at the longer (*M* = .79, *SE* = .022) as opposed to the shorter (*M* = .62, *SE* = .22) presentation time. Furthermore, as predicted by the ABC hypothesis, there was an interaction between presentation time and stimulus congruency, with a larger effect at the shorter interval, *F*(1, 19) = 7.36, *p* = .014, η_p_^2^ = .28. As illustrated in Fig. 2, the increase in presentation time effectively halves the congruency effect (difference short presentation = .37, difference in longer presentation = .18).

### Discussion

We proposed that congruency effects are due to stimulus control introducing processes that direct attention to nonnumeric stimulus characteristics and bias stimulus selection toward more salient stimuli. Under the short presentation times that often are used in dot comparison tasks, salient nonnumeric stimulus characteristics might be particularly prone to divert participants’ attention from the relevant dimension of numerosity, while leaving little time to correct the initial judgment. In support of our hypothesis, we found that the congruency effect was reduced by a longer presentation time. It has been argued that visual nonnumeric cues are flexibly integrated on a trial-by-trial basis to extract estimates of numerosity (Gebuis & Reynvoet, 2012). Thus, cue integration might operate differently depending on whether brief or long presentation times are used. However, such an overly flexible cue-integration account is close to impossible to falsify.

Although the results so far support the attention account, we do not have any direct measure of attention in the task, and it is possible that some other process is responsible for congruency effects. In Experiment 3, we measured attentional processes directly using eye-tracking. This method allows a fuller understanding of attentional processes during the early phases of a dot-comparison task (Castaldi et al., 2020). We predicted that participants would be more likely to direct their first gaze to the stimulus with the dots of larger individual size. For congruent stimuli, this would mean that the first gaze shift would be to the more numerous stimulus set. For incongruent stimuli, the first gaze shift would be towards the less numerous stimulus set, biasing participants to choose this stimulus as the more numerous array more often. Again, we relied on a commonly used method of stimulus creation (Gebuis & Reynvoet, 2011) in order to generalize conclusions to attentional processes directly relevant to previously conducted research.

## Experiment 3

In Experiment 3, participants carried out a dot-comparison task while we measured their gaze using an eye-tracker (cf. Castaldi et al., 2020; Odic & Halberda, 2015). We predicted that attention would initially be directed to the array with dots of larger individual size and that participants would have little time to carry out any additional, corrective eye-movements under the short viewing conditions employed.

Furthermore, we expected participants to judge the first fixated array as the more numerous one. Previous research has indicated that using eye-tracking makes it possible to gain a deeper understanding of how stimulus in dot-comparison tasks are processed and encoded (Castaldi et al., 2020; Odic & Halberda, 2015), suggesting that it might also be a useful tool to study attentional processes.

### Methods

#### Participants

Participants (40, 17 males, 23 females, *M*_age_ = 24.2 years, *SD*_age_ = 5.44 years) were mainly undergraduate students at Uppsala University. All gave informed consent to participate and received either a cinema voucher or course credits for participating.

#### Apparatus and stimuli

Gaze was recorded with a Tobii T120 (Stockholm, Sweden) eye-tracker at 60 Hz while the stimuli were presented on a 17-in, screen with a 1,280 × 1,024resolution. The stimuli consisted of 90 images containing two spatial separated arrays of black dots on a white background. As in Experiment 2, the images were created using the MATLAB script provided by Gebuis and Reynvoet (2011). Because we were interested in the effect of stimulus congruency on attention rather than ANS acuity per se, all images had a 7:8 ratio between the numerosity of the two arrays. Half of the images had seven and eight dots in the two arrays, while the other half had 14 and 16 dots. Stimulus congruency was manipulated within subjects. One-third of the images were *congruent,* with the more numerous array also consisting of larger dots (average radii 2.5 times larger) and a larger convex hull angle (see Table 1 for further details about stimulus set characteristics). Another third of the images were *incongruent,* with the less numerous array having larger dots (average radii 1.8 times larger) and a larger convex hull. Finally, one-third of the images were *neutral,* where no systematic difference between two arrays in terms of dot size was introduced. The diameter of the dots varied between .18 and 1.26 visual degrees, and they were presented in an area subtending approximately 10.5 × 10.5 visual degrees. The centers of the arrays were separated by approximately 20 visual degrees.

#### Procedure

Participants were seated approximately 60 cm in front of the eye-tracker. They were instructed to remain as still as possible during the entire procedure. Before the experimental procedure, a 5-point calibration was performed (Gredebäck, Johnson, & von Hofsten, 2009). This procedure was repeated until a satisfactory calibration could be achieved. Following the calibration procedure, participants completed five (5) training trials followed by 90 test trials. On each trial, participants were first presented with a fixation cross at the center of the screen for 500 ms, followed by the dot stimuli presented for 1,000 ms. After the stimulus presentation, participants made a verbal response as to which array was more numerous. Note that in contrast to previous studies, participants were not explicitly instructed to shift their gaze towards the more numerous stimulus set (Castaldi et al., 2020). The verbal response was recorded by an experimenter. The stimuli were presented in one of two fixed orders, counterbalanced over participants.

#### Data reduction and analysis

Gaze data were exported from the eye-tracker as fixation data (Tobii fixation filter, Velocity threshold = 35 pixels/window, distance threshold = 35 pixels) and imported into the open-source analysis framework TimeStudio 3.16 (Nyström, Falck-Ytter, & Gredebäck, 2016) running in MATLAB 8.4.

We analyzed participants’ gaze towards three areas of interest (AOIs), one centred at the middle of the screen and the other two cantered over the two dot arrays, respectively. The centre AOI measured 7.6 × 11.3 visual degrees, while the AOIs over the two dot arrays measured 11.3 × 11.3 visual degrees. We calculated two dependent measures: the looking time towards each of the three AOIs during the presentation of the dot arrays, and which of the two dot array AOIs participants directed their first gaze towards after stimulus onset. In an effort to reduce the impact of very short saccades and quick, corrective saccades (i.e., an initial saccade towards one stimulus array, which is then immediately corrected towards the other stimulus array), we operationalized the first gaze as the first AOI in which participants had a valid fixation, as determined by the fixation filter used.

## Results

**Fig. 3:**
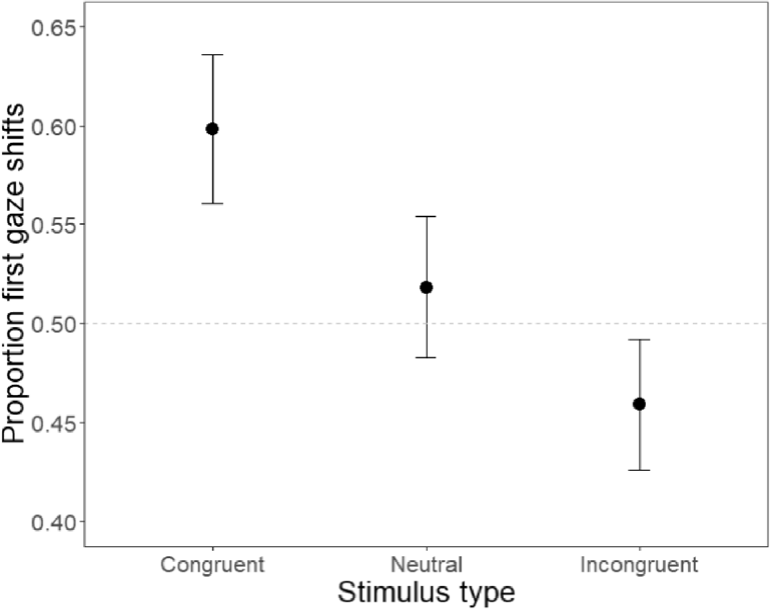
Proportion first gaze shifts towards the more numerous stimuli as a function of stimulus type. Whiskers denote 95% confidence intervals, and the dashed grey line denotes chance performance at .5

We compared the proportion of the first gaze towards the more numerous dot array for the three types of stimuli (congruent, incongruent, and neutral) with a one-way, within-subjects ANOVA. This analysis, illustrated in Fig. 3, revealed a significant effect of stimulus type, *F*(2, 78) = 25.9, *p* < .001 η_p_^2^ = .40. As predicted, participants were more likely to direct their first gaze toward the more numerous stimuli on the congruent than on the incongruent stimuli. Follow-up post hoc tests (Scheffe’s) showed that performance on all three stimulus types was significantly different (*p*s < .015) from each other. For congruent stimuli, participants were more likely to direct their first gaze towards the more numerous stimuli than can be expected by chance, *H*_*0*_ = .5, *M* = .60, 95% CI [.56, .64], *t*(39) = 5.31, *p* < .001, while the opposite was true for incongruent stimuli, *H*_*0*_ = .5, *M* = .46, 95% CI [.43, .49], *t*(39) = 2.50, *p* = .017. With neutral stimuli, participants were equally likely to direct their gaze towards the more as towards the less numerous array, *H*_*0*_ = .5, *M* = .52, 95% CI [.48, .55], *t*(39) = 1.04, *p* = .31.

We calculated the proportion of trials in which the stimulus that participants directed their first gaze towards was also the stimulus they chose. If the early shift in attention is unrelated to participants’ choices, this proportion can be expected to be .5. A single-sample *t* test indicated that this proportion was significantly larger than .5, *H*_*0*_ = .5, *M* = .54, 95% CI [.52, .56], *t*(39) = 4.21, *p* < .001. Thus, participants were more likely to choose the array they directed their first gaze towards. We evaluated the effect of this response bias by investigating whether a first gaze towards the more numerous stimulus was associated with a higher likelihood of a correct response. We compared the proportion of correct responses when the first gaze had been directed towards the more numerous array with the proportion of correct responses when the first gaze had been directed towards the less numerous array. This analysis revealed a significant difference, *t*(39) = 2.42, *p* = .02, with a higher proportion of correct responses (*M* = .73, *SD =* .11) when the first gaze was directed towards the more numerous array than when it was directed towards the less numerous array (*M* = .67, *SD* = .12).

We investigated whether the proportion of looking time towards the more numerous stimulus was different over stimulus types, only including trials where participants looked at one or both of the two stimuli. The dependent variable was calculated by dividing the looking time towards the more numerous array with the total looking time to all three AOIs (middle AOI and the two dot-array AOIs). This measure was submitted to a within-subjects ANOVA, with the proportion of looking time towards the more numerous AOI as a dependent measure and stimulus type (congruent/incongruent) as the independent, within-subjects factor. The analysis showed a nonsignificant effect of stimulus type *F*(1, 39) = 1.78, *p* = .19, *η*^*2*^_*p*_ = .04 revealing that participants spend an equal proportion of looking time towards the more numerous array for both congruent (*M* = .37, *SD* = .05) and incongruent (*M =* .38, *SD* = .04) stimuli.

## Discussion

Participants’ attention was influenced by stimulus type. More specifically, as predicted, they were inclined to direct their first gaze to the dot array with dots of larger individual size. Thus, for incongruent stimuli, they first looked at the less numerous stimulus set, but for congruent stimuli, they first looked at the more numerous stimulus set. This finding is in line with previous research indicating that attention is driven in a bottom-up fashion by both the size of the stimuli (Wolfe & Horowitz, 2004) and the number of objects in the stimulus set (Castaldi et al., 2020), but extends this research by showing that these two properties compete for attention.

Participants were more likely to give a correct answer when their first gaze was directed towards the more numerous array than when it was directed towards the less numerous array. Nevertheless, the behavioral choice data should be evaluated with caution because these were obtained after a longer presentation time than usual and were given verbally to the experimenter, rather than through a rapid motor response. In line with our ABC hypothesis, we find it reasonable to conclude that involuntary rapid bottom-up attention effects are a contributing cause of congruency effects (Gilmore et al., 2013; Szűcs et al., 2013).

The overall looking time towards the stimulus sets did not vary over stimulus type, however. This suggests that the effect is not due to this variable, but that the first gaze is crucial. It should be noted, however, that using eye-tracking forced us to use a longer presentation time (1,000 ms) than is ordinarily used in ANS research (around 300 ms). With shorter presentation times, it is unlikely that participants will have time to make a switch to the opposite stimuli. Thus, they may even end up only focusing on the congruent stimuli on any single trial, which would not allow them to correct their initial response.

## General discussion

Humans are believed to be equipped with a nonverbal cognitive system for extracting the number of objects in a visual scene (Feigenson et al., 2004), the ANS. Previous research has indicated a relation between the acuity of the ANS and math performance (Schneider et al., 2017) and adults (Chen & Li, 2014). However, the results are far from consistent, and recently, several studies have not been able to find the ANS-to-math-performance link (e.g., Inglis et al., 2011). One possibility is that humans lack a dedicated system for number processing altogether (Gebuis & Reynvoet, 2012; Leibovich et al., 2017). In line with this possibility, some studies have found that when controlling dot stimuli for properties other than number (e.g., average area, convex hull), the correlation between math performance and ANS acuity disappears (Gilmore et al., 2013). Importantly, controlling for visual cues in such a way that half of the stimuli are congruent and the other half are incongruent results in strong congruency effects where participants perform close to, or even below, chance on incongruent trials (Szűcs et al., 2013).

An alternative account for the strong congruency effects is an attentional bias induced by stimulus control, ABC, that arise when using extreme measures to control for visual, nonnumerical cues. This manipulation introduces attentional processes that dominate or distract participants from extracting numerosity. Strict stimulus control might contaminate measures of ANS acuity and make them less valid. In the current study, we designed three experiments to test this possibility.

In Experiment 1, we tested the first predictions of the ABC hypothesis. Participants carried out two dot-comparison tests, representing two possible measures of ANS acuity. For the first, we introduced stimuli that were neutral regarding the preattentive dimension of object size by equating this size in the to-be-compared arrays. Like what could be expected in a natural setting, this allows variables such as total area, convex hull, and density of the stimulus to covary with numerosity. For the second test, we instead used a deliberate manipulation of object size with stimuli divided into congruent and incongruent regarding this dimension, analogous to the resulting size difference induced by rigorous stimulus control.

While performance on the neutral test was correlated with most math-related variables, performance on the size-manipulated test was not. Within the incongruent and congruent subsets of the size-manipulated test, only the incongruent (but not the congruent) subset predicted math variables. This pattern was predicted and mimics results in previous research. Furthermore, the size-manipulated test did not exhibit internal consistency. Performance on the congruent and incongruent stimuli was negatively correlated, and performance on the incongruent stimulus was uncorrelated with total performance on the task. The stimuli used in the first experiment rely on a mere manipulation of size and are not identical to those used in previous ANS research. The claim is not that this study demonstrates that standard control measures would necessarily be subject to the same effects, but the fact that such controls routinely incorporate size differences between stimuli should in itself raise concerns. The results are in line with the hypothesis that using extreme controls for visual cues in dot-comparison tests may contaminate them by introducing additional processes.

The results of Experiment 1 do not speak to what processes might cause congruency effects. Experiment 2 was designed to investigate the cause of these effects. If attention processes are behind congruency effects, these should be reduced with more time to process the stimuli and counteract initial, rapid, bottom-up attention. According to the notion that participants are not using a dedicated system for extracting numerosity, but are guided entirely by nonnumeric cues when estimating number, we should expect the opposite. We found a congruency effect that was reduced by a prolonged presentation. This result supports the idea that congruency effects can be accounted for by attentional processes, but it seems inconsistent with an account suggesting that people fundamentally extract numerosity by means of integrating visual cues.

Experiment 3 addressed the concerns of a lack of a direct measure of attention. Results showed that stimulus type had an effect on which dot array participants directed their initial attention to. They predominantly looked first towards the stimulus with the dots of larger individual size. For congruent stimuli, they consequently initially looked at the more numerous array, but for incongruent stimuli, they looked towards the less numerous array. The stimulus that participants looked at first was associated with their responses, so that when they looked at the less numerous stimulus set first, they were more likely to be wrong than when the initial gaze was towards the more numerous stimulus set.

These results suggest that nonnumeric stimulus properties drive participants’ attention, which in turn influences their responses. It is reasonable to conclude that the short presentation times often used in dot-comparison tasks do not allow participants to correct an initial bias to respond in accordance with the first fixated array.

There is by now a large body of evidence showing that nonnumeric variables influence number estimation (e.g., Gebuis & Reynvoet, 2012; Gilmore et al., 2013; Poom et al., 2019; Szűcs et al., 2013; Tokita & Ishiguchi, 2010). Thus, a naïve notion of an approximate number system that allows us efficiently to estimate number, irrespective of the circumstances, can hardly be defended. However, the numerous demonstrations that judgments of numerosity are influenced by nonnumeric variables should not be taken as evidence that nullify the existence of a dedicated system for numerosity processing. It is rather a general phenomenon in perception that visual experiences of basic features, such as color and motion, are influenced by a number of variables other than the activation of dedicated feature-specific detectors. For example, a basic visual task, such as color perception, depends on context (simultaneous color contrast), the spectral content of the illuminating light which is discounted (allowing color constancy), whether or not the colored target is in shadow or directly illuminated (allowing brightness constancy), adaptation with subsequent color aftereffects, prior experiences with objects of known color (leading to biases in color matching tasks), and synaesthesia, where for some people input in other sensory organs are misperceived and appear as colors. These cues modify the perceptual experience and modify the output from the excitations of the three types of cones acting as bandpass filters of the spectra of incoming light in the retina. However, despite the influence of such extraneous variables on color perception, there can be no doubt that humans are equipped with a system dedicated to color perception.

There are, of course, limitations to the current study. Numerosity and nonnumerical stimulus properties are necessarily coupled. We have argued that item size may be the crucial preattentive dimension, but array area and convex hull may also be involved in preattentive attraction of gaze, something that cannot be disentangled from the results in Experiment 3 since these variables covaried. In Experiments 1 and 2, there is a confound between item size and the proportion of covered area within the array, since the array area was kept constant in congruent and incongruent subsets. Still, since item size has been found to be a stimulus feature picked up preattentively, this stimulus dimension is, in our opinion, most likely the culprit in this case (Wolfe & Horowitz, 2004). It should be noted that number is also a feature that can be picked up preattentively (e.g., Castaldi et al., 2020; Cicchini et al., 2016; Ferrigno et al., 2017). Thus, it is likely that item size becomes relatively more salient than the number of objects in incongruent stimulus sets, while the two dimensions align in congruent stimulus sets.

Our results show that overt reflexive attention, defined as selectively processing one location over others by moving the eyes to a point at that location influences decisions in size-manipulated numerosity tasks. Covert attention, defined as paying attention without moving the eyes (Posner, 1980), may nevertheless also play a role here since both types of attention behave similarly (Blair & Ristic, 2019). Most of the studies that find a link between math proficiency and ANS in adults have used spatially intermixed dot displays, whereas failures to demonstrate a link often have used spatially separated displays (see Norris & Castronovo, 2016). This could be interpreted as overt attention and gaze playing more important roles in separated displays that therefore should be avoided. However, spatially intermixed presentation is probably no safeguard against attentional effects. Both congruency effects and the pattern of correlations where performance on incongruent items drives correlations with math have been found with intermixed presentation (Norris & Castronovo, 2016), implying that covert attention processes play a role also with the latter design. In addition, as spatial based attention is directed to locations in the visual field, object-based attention is directed to organized chunks of visual information corresponding to an object or a group of items belonging to the same object or a group in the environment (Mozer & Vecera, 2005). Potentially, both such object-based attention and covert attention processes could account for influences of preattentional features when using spatially intermixed displays.

A methodological implication of these results would seem to be that applying a sequential one-at-a-time stimulus presentation would be preferable because this presentation eliminates attentional processes. However, as shown by Lindskog et al. (2013), sequential presentation may be less predictive of math performance due to the introduction of a time-order error by which the most recently presented stimulus set appears to be more numerous (the “recent-is-more” effect; see van den Berg, Lindskog, Poom, & Winman, 2017), that further complicates the use of this method.

Researchers have repeatedly emphasized the importance of controlling for visual variables in ANS acuity number estimation tasks and have taken great measures to achieve this. This notion is echoed in a methodological review (Dietrich, Huber, & Nuerk, 2015), where it is stated that “controlling visual properties is essential to ensure that the task really measures the ability to discriminate numerosity and not the ability to discriminate visual cues” (p. 10). Our results imply that this appeal for control does not come without serious caveats. We surmise that some studies with a zealous aim to achieve this control paradoxically have ended up with stimuli that, in an almost grotesquely conspicuous way, have signalled nonnumeric visual properties such as object size, with a strong impact on judgment processes. Often, these studies, rather than “controlling for” visual properties are better described as psychological experiments set up to determine whether or not number judgments are influenced by nonnumeric continuous visual properties when these are manipulated systematically. That nonnumeric variables influence number judgment is a well-established fact, albeit not entirely surprising. The message conveyed here is that maybe we should direct our effort at more interesting theoretical research problems other than how to cleverly achieve stringent stimulus control and consequently having to explain congruency effects that are laboratory artifacts of this control. It is by now clear that control of nonnumeric features can create large problems when measuring ANS acuity, but it is less obvious what, if any, problem that actually is solved by this procedure. If we are worried by participants relying on nonnumeric attributes, we should obviously instead refrain from controlling for these variables altogether, because, as we have shown, such control has the opposite effect. If a participant, when making a number estimate, is influenced by, for example, the cumulative area of the objects, just like he or she could be in a natural environment, we would not necessarily consider this a problem.

In a natural environment, certain statistical regularities between perceptual variables hold. Organisms have evolved to exploit these regularities, and they should be preserved in the laboratory, instead of being distorted by rigorous control. The fact that cumulative area commonly covaries with number is not a bug, but a feature of the environment. In accordance with Brunswik (1955), we argue for the necessity of a design that is representative of the environment and, instead of interfering with this environment, strives to retain its causal texture in stimuli that are used in the laboratory (see Dhami, Hertwig, & Hoffrage, 2004, for an extensive elaboration of this “representative design” concept). However, rigorous stimulus control is reasonable to use in investigations aimed to discover whether infants or animals can discriminate between numerical and nonnumerical stimulus dimensions, or have any concept of numerosity at all (Feigenson et al., 2004). Thus, stimulus control may be necessary when asking the existence research question “Can they do this?,” but could be counterproductive in measuring proficiency in the ability: “How well does individual X perform at this?”

To summarize, results from the experiments provide support for the idea that attentional processes can account for congruency effects in dot-comparison tasks. There are also two further implications from our findings. First, even though it is commendable that researchers want to control strictly for possible confounds in dot-comparison tasks, it is important to note that when such controls are made in an extreme way, they most likely coincidently contaminate the tasks, leaving them less valid measures of ANS acuity. Second, although much more research is needed before the debate is settled, congruency effects per se cannot be taken as evidence that humans lack a dedicated system for extracting numerosity from a visual scene.

## Footnotes

1 We use accuracy rates as dependent measure of performance because this measure is straightforward and assumption-free regarding the underlying processes. Estimating Weber fractions (*W*) would rely on assumptions that are expected not to hold here, with dominating processes of attention and systematic bias, rather than sensitivity to number as modelled by the psychophysical model. In addition, two papers (Inglis & Gilmore, 2014; Lindskog et al., 2013) investigating psychometric properties of dependent variables of ANS both advocate use of accuracy. However, the predictions are not contingent on a particular dependent measure, and high correlations between *W* and accuracy have been found (e.g., Lindskog et al., 2013 reported *r* > .95).

2 As a comparison, in the neutral test, there was as expected a significant positive correlation between two randomly selected test halves made up of stimuli of identical ratios, *r*(56) = .60, *p* < .001, indicating a split-half reliability of .75, as estimated with Spearman–Brown prophecy formula.

3 The single-order correlation between the full size-manipulated test performance and performance on the incongruent subset is not significant because it is suppressed by the negative correlation between performance on the two subsets. Accordingly, controlling for performance on the congruent subset reveals a significant partial correlation between performance on the incongruent subset and full size-manipulated test performance, *r*(56) = .59, *p* < .001.
